# Dissertation on Medical Delusions

**Published:** 1851-05

**Authors:** 


### ARTICLE II.

*Fisk Fund Prize Desertation of the Rhode Island Medical Society.*

*Lessons from the History of Medical Delusions*, By WORTHINGTON HOOKER, M. D., author of "Physician and Patient."

"*Falax non raro experientia, si Rationis ductû fuerit destituta. Qua propter nisi mutuam sibi lucem communicent, æquam erroris causam præbebunt.*"—*Baglivi.* New York, Baker & Scribner, 1850; pp. 105, 12 mo., (from A. H. & C. Burley.)

Just at this time, this little work is exceedingly opportune. Perhaps it might have been as much needed at any other point of time in medical history, but we appreciate the present more forcibly as a time when the people are disposed to run after the medical lo! heres and lo! theres, than any other period of our professional career.

Although we think Homœopathy is beginning to wane in public estimation, and Hydropathy not likely to prevail to any thing like the same extent, there will undoubtedly be some other form of delusion to take their places when they have passed away. In fact it is a long time before any popular delusion will entirely die away. There are yet a few steam doctors left, of the host that a few years ago threatened to take the faith of the entire public to themselves. We are right glad that a moderate, philosophical, and fair exposition of the foundations of medical delusions has been made in the pleasing and elegant style of our author.

Most of the errors leading to these delusions are the result of losing sight of the power of nature to cure, or in other words, in attributing to pretended remedies what legitimately belongs to the *vis medicatrix natura.*

Our author illustrates this position by many examples, a few of which we cannot refrain from giving in his own language, though the facts may be familiar to many of our readers.

Bishop Berkley's tar-water-drinking delusion is related, with Luther's assertion, that:

"Experience has proved the toad to be endowed with valuable qualities. If you run a stick through *three* toads, and after having dried them in the sun apply them to any pestilent tumor they draw out all the poison, and the malady will disappear."

Our author continues:

"One of the most successful of medical delusions was broached by a physician in Connecticut. I refer to Perkins' Tractors. The Tractors were two pieces of metal, one appearing to be steel, and the other brass, about three inches in length and tapering to a point. Their efficacy, it was supposed by the inventor, and by the many learned men who endeavored to account on philosophical principles for the effects, which they were almost universally believed to produce, depended upon the developement of galvanic fluid.

Dr. Perkins' discovery was promulgated in 1796. In two years the Tractors were introduced into England, and some other European countries. In eight years Perkinism, as it was called, was so triumphant, that a Perkinean institution was formed in London, with a large proportion of its members from among the ranks of the titled, the learned, and the reverened. The Tractors, which readily sold for five shillings a pair,* were under the auspices of this institution applied most benevolently to the sick and suffering poor. This society had its public dinners in honor of the grand discovery, and poetry even was laid under extensive contribution to sound its praises and diffuse its benefits. Thus runs the strain of one of the Perkinean poets:

"See pointed metals blest with power to appease
The ruthless rage of merciless disease,
O'er the frail part a subtle fluid pour,
Drenched with invisible galvanic shower,
Till the arthritic staff and crutch forego,
And leap exulting like the bounding roe."

The Perkinean committee published from time to time their reports of the cures, which as early as 1802 were stated to number five thousand. Some of the certificates, many of which were from the highest sources, were of the most decisive and enthusiastic character. A divine, a professor in one

*The Tractors were manufactured in a small village, near the author's place of residence, for *one shilling* a pair.

of our New England Colleges, thus wrote: "I have used the Tractors with success in several other cases in my own family, and although, like Naaman the Syrian, I cannot tell why the waters of Jordan should be better than Abana and Pharpar, rivers of Damascus; yet since experience has proved them to be so, no reasoning can change my opinion." Volumes of these certificates were published in succession, and Perkinism was proclaimed as the grand medical discovery of the age.

And yet, in seven years from the founding of the Perkinean institution, so changeable is the popular medical mind, the Tractors were spoken of as being almost forgotten; and at the present time it is almost impossible to find a pair of them as relics of a past folly, and if the offer should be made of the old price of five guineas, it would bring but a few of them to light.

Now the same error was committed by the believers in the Tractors which was made by Bishop Berkley in regard to the Tar Water. They forgot that the curative power of nature is always at work removing disease, and that imagination sometimes renders essential assistance. They thought that all who got well after the application of the Tractors were cured by the Tractors, as Berkley did that all who got well after swallowing Tar Water were cured by Tar Water.

"There were unbelievers in the days of Perkins, as there were in the days of Bishop Berkeley; and they were wicked enough to try experiments on their patients with Tractors made of wood, and painted so as to resemble the five guinea Tractors. They were impudently pretended to produce the same effects, and five of the patients of these mischievous doctors returned public thanks in church for their cures. In one of these cases of cure, effected by the wooden Tractors, the patient, Miss Ann Hill, after a little time exclaimed, 'Bless me, why, who could have thought it, that them little things could pull the pain from one. Well, to be sure, the longer one lives, the more one sees; ah, dear!' And if Miss Ann Hill had lived in this year of grace, 1850, she would have had her pain drawn out by the hands of a professor of animal magnetism, or pyschology, as it is now called, instead of painted wooden Tractors."

The author makes the following application of his reasoning to the present popular delusion of infinitesmal medication.

"The error which I have been illustrating is carried to an extreme by the Homœopathist. He attributes palpable results to doses of medicine which are so small that they cannot produce any perceptible effect except by miracle. Can it be seriously believed that a decillionth of a grain of charcoal, or oyster shell, or common salts will produce manifest effects in the system? And yet this is what Homœopathy asserts.

"The experience upon which such assertions are based is acquired in a very singular way. A person takes one of these little doses, of oyster shell for example, the effects of which are said to last fifty days. All the symptoms which he has during that time are to be put down, if a record be kept of his case, as the effects of that oyster shell. Among the notes thus made will perhaps be the following: After dinner, disposition to sleep; the patient winks; tremor of the hands when occupied with fine, small work; the upper lip becomes cracked; phlegm is hawked out, chiefly in the morning; there is a voluptuous tickling on the sole of the foot after scratching; a little indolence, aversion to talk; joylessness and disinclination to labor; attacks of anxiety, especially at evening; inflammation and swelling of one-*half* of the nose; an itching, tickling sensation at the *outer* edge of the *palm* of the *left* hand, which obliges the person to scratch; crooking of the fingers *when gaping*, and cramp in them at *midnight;* cool perspiration of the hands, frequently with a *cold point of the nose;* boring, grubbing tooth-ache, increased by mental exertion; thirst, with loathing of *water and beer;* anxious hesitation and disconsolateness with *reflections on death;* twitching in the cartilage of the ear, and pricking *behind* the ears; creeping in the *upper* lip and in the *point* of the nose; on awaking the *right* arm over the head; awakes with perspiration and heat at *three o'clock* in the morning; walks with a self-sufficient importance; when stepping out walking a sensation on the *back* of the foot as if the boot were too tight; the little toe aches as if hard pressed; burning near a *golden* ring on the *fore* finger; drawing pain on the head when brushing the hair *backwards;* tightness in the *small* toe of the *left* foot, &c.

"This is no caricature of the Homœopathic mode of recording cases. These are actual quotations from a standard work on Homœopathy, a closely printed octavo volume of 600 pages, purporting to be an arranged collection of the observations of Homœopathic physicians, in regard to the operation of substances upon the system, both in health and disease.

The most wild and fertile imagination, set loose from reason, to roam where it listeth, could not collect a more incongruous and ridiculous farrago, than is to be found in Jarh's Manual, under the guise of scientific observations of the effects of remedies. With the Homœopathist, the infinitesimal dose is everything; all else, even the universe with all its agencies great and small, is nothing. His favorite antecedent, though Allopaths may call it tiny, neutralizes, or swallows up, all other antecedents, by its magic 'dynamic power.'

"You observe that the infinitesimal doses are proved to cure disease, precisely as Perkin's Tractors, the Weapon Ointment, and the Tar Water of Berkeley, were once proved to do it. The reasoning is this: A patient took a decillionth of a grain of oyster-shell three or four times a day; he got well; *therefore* the oyster-shell cured him."

But we cannot pursue the analysis of the work any further. It shows up the errors of the regular profession, as well as the gross follies of the quacks and the simpler folly of the people in following their delusions, in a clear and interesting manner; so that if the work could be generally read, the professional man would be better able to explain the devices of the special system men to gain the public confidence, while the public might be better prepared to judge who were the true philosophers by having their philosophy expounded.

Some of our contemporaries are disheartened with efforts to correct the errors and follies of pretenders and the public in reference to medical matters, because they say quackery cannot be got rid of, and hence they speak of such works as the one before us, as being well enough, but not likely to do much good. Now our doctrine is, that in the great contest between truth and error every successful effort on the part of truth is so much of clear gain, notwithstanding we may never be able to eradicate all the error in the world.

We therefore most cordialy recommend the little work before us to the perusal of all enquirers after medical truth, confident that it will have a salutary influence wherever read in leading to a better estimation of the profession of medicine.

